# Epigenetic alternations of microRNAs and DNA methylation contribute to gestational diabetes mellitus

**DOI:** 10.1111/jcmm.15984

**Published:** 2020-10-21

**Authors:** Weiqiang Zhu, Yupei Shen, Junwei Liu, Xiaoping Fei, Zhaofeng Zhang, Min Li, Xiaohong Chen, Jianhua Xu, Qianxi Zhu, Weijin Zhou, Meihua Zhang, Shangqing Liu, Jing Du

**Affiliations:** ^1^ Key Laboratory of Birth Regulation and Control Technology of National Health Commission of China Shandong Provincial Maternal and Child Health Care Hospital Affiliated to Shandong University Jinan China; ^2^ NHC Key Lab. of Reproduction Regulation Shanghai Institute of Planned Parenthood Research) Pharmacy School Fudan University Shanghai China; ^3^ The First people's Hospital of Kunshan Kunshan China; ^4^ Department of Obstetrics and Gynecology Shanghai Pudong New Area Health Care Hospital For Women & Children Shanghai China; ^5^ North Sichuan Medical College Nanchong China

**Keywords:** DNA methylation, epigenetics, gestational diabetes mellitus, microRNA

## Abstract

This study aimed to identify epigenetic alternations of microRNAs and DNA methylation for gestational diabetes mellitus (GDM) diagnosis and treatment using in silico approach. Data of mRNA and miRNA expression microarray (GSE103552 and GSE104297) and DNA methylation data set (GSE106099) were obtained from the GEO database. Differentially expressed genes (DEGs), differentially expressed miRNAs (DEMs) and differentially methylated genes (DMGs) were obtained by *limma* package. Functional and enrichment analyses were performed with the DAVID database. The protein‐protein interaction (PPI) network was constructed by STRING and visualized in Cytoscape. Simultaneously, a connectivity map (CMap) analysis was performed to screen potential therapeutic agents for GDM. In GDM, 184 low miRNA‐targeting up‐regulated genes and 234 high miRNA‐targeting down‐regulated genes as well as 364 hypomethylation–high‐expressed genes and 541 hypermethylation–low‐expressed genes were obtained. They were mainly enriched in terms of axon guidance, purine metabolism, focal adhesion and proteasome, respectively. In addition, 115 genes (67 up‐regulated and 48 down‐regulated) were regulated by both aberrant alternations of miRNAs and DNA methylation. Ten chemicals were identified as putative therapeutic agents for GDM and four hub genes (IGF1R, ATG7, DICER1 and RANBP2) were found in PPI and may be associated with GDM. Overall, this study identified a series of differentially expressed genes that are associated with epigenetic alternations of miRNA and DNA methylation in GDM. Ten chemicals and four hub genes may be further explored as potential drugs and targets for GDM diagnosis and treatment, respectively.

## 1 INTRODUCTION

Gestational diabetes mellitus (GDM) is defined by ‘the type of glucose intolerance that develops in the second and third trimester of pregnancy, resulting in hyperglycaemia of variable severity’.^1^ GDM affects 2%–5% of pregnancies worldwide, with the significantly increased prevalence over the last decade.^2^ Effects of GDM involve dysfunction of glucose metabolism and an increase of glucose accessibility by the foetus, which cause adverse pregnancy results or negatively impact the health of both mother and foetus. Moreover, GDM is related to adverse consequences not only during foetal development of pregnancy but also later in life.^3^, ^4^ Many literatures indicated genetic, epigenetic or environmental factors can contribute to GDM.^5^ Moreover, increased insulin resistance stimulated by a genetic predisposition to impairment of pancreatic islet β‐cell function may also contribute to GDM during pregnancy.^6^ In the human umbilical vein and placental microvascular endothelia, GDM may also associate with foetoplacental vascular dysfunction characterized by increased NO synthesis and L‐arginine transport activity.^7^ While knowledge concerning the detailed processes governing the initiation and progression of GDM is still unknown and remains a key obstacle on the road of GDM treatment, the development of robust and accurate biomarkers will greatly facilitate the early detection and identification of biological features of GDM. Therefore, more potential biomarkers and some chemicals for GDM are urgent to be identified.

Currently, many studies suggested that epigenetic modification can play an important role in the pathogenesis of some multifactorial diseases, including GDM.^8^, ^9^ The epigenetic modification includes histone modification, DNA methylation and miRNA gene silencing. All of them are closely linked with each other and influence protein synthesis patterns.^10^ Disturbance of this complementary system may lead to dysfunction.

MicroRNAs are highly conserved small single‐stranded non‐coding RNA (18 ~ 25 nucleotides; nt) post‐transcriptionally modulating gene expression in a variety of disease‐related signalling processes and pathways.^11^ They have emerged as promising diagnostic and therapeutic tools due to their association with GDM. For instance, miR‐137 displays high expressions, whereas its target gene FNDC5 (fibronectin type III domain containing 5) is down expressed between women with GDM and normal in the placenta tissues, which can restrict the viability and migration of trophoblast cells.^12^ Among the numerous aberrantly expressed miRNAs discovered in patients with GDM, miRNA‐340 down‐regulated PAIP1 (poly(A) binding protein‐interacting protein 1), which involved in glucose and insulin regulation.^13^

It has also been found that a low level of miR‐21‐3p in blood leucocytes of women may increase the risk of GDM.^14^ In addition, associations of miR‐21‐3p with GDM were present only among women carrying male foetuses.^15^

Furthermore, 32 different types of miRNA have been identified, differentially expressed in GDM women plasma compared to non‐GDM women. Most of aberrantly expressed miRNAs are associated with glucose and insulin metabolism by affecting disrupting the MAPK (mitogen‐activated protein kinase) signalling pathway or IRS genes.^16^ Ding R, et al found miR‐138‐5p differentially expressed in GDM mouse placentas and significantly inhibited the proliferation and migration of trophoblasts (HTR‐8/SVneo) by targeting the 3'‐UTR of TBL1X (transducin beta‐like 1X‐linked) gene.^17^ Tang X‐W, et al reported miR‐335‐5p suppressed pancreatic islet β‐cell secretion and enhanced insulin resistance by inhibiting VASH1 (vasohibin 1) in GDM mice, eventually activating the TGF‐β pathway.^18^

Aberrant DNA methylation, another common and best‐studied epigenetic modification, makes a critical contribution in the regulation of genomic imprinting, genome stabilization gene expression and chromatin modification, which engage in placental development.^19^ Numerous studies have revealed that aberrant DNA methylation has been reported to be engaged in adverse pregnancy outcomes, including GDM.^3^ A study revealed that 56 pregnant women with GDM displayed a significant increase of global placental DNA methylation compared with 974 controls, independent of risk factors such as maternal age, BMI and recurrent miscarriages.^20^ During the stages of embryogenesis, LEP (leptin) methylation profile was increased in placentas and umbilical cord blood in GDM.^5^ Interestingly, DNA methylation of the LEP gene was evaluated in foetal tissues and associated with glycaemia after two hours of an oral glucose tolerance test (OGTT).^21^ On the contrary, some genes with lower DNA methylation levels also take part in the GDM process. For instance, lower DNA methylation levels in the promoter of ADIPOQ on the foetal side of the placenta were correlated with higher maternal glucose levels and ADIPOQ is suspected to have insulin‐sensitizing proprieties.^22^ Besides, three gene GLUT3 (Glucose transporter 3), Resistin and PPARα (peroxisome proliferator‐activated receptor alpha) showed significant hypomethylation in GDM compared to control subjects, all of them have potential functions with energy metabolism in pregnancy.^3^

Until now, although multiple studies have demonstrated the global methylation level and microRNA level, or certain genes with aberrant DNA methylation and expression level in GDM, the comprehensive regulatory network and pathways have not been profiled about how epigenetic alternations of microRNAs and DNA methylation are related to GDM.

In this study, data of mRNA expression profiling microarrays (GSE103552), miRNAs expression microarrays (GSE104297) and DNA methylation microarrays (GSE106099) were systematically analysed in order to identify core genes and pathways which contribute to GDM via epigenetic regulation.

## 2 METHOD

### 2.1 Microarray data

In this present study, data of mRNA and miRNA expression profiling microarray (GSE103552 and GSE104297) and gene methylation profiling data sets (GSE106099) were retrieved and obtained from the GEO database (Gene Expression Omnibus, https://www.ncbi.nlm.nih.gov/geo/) in the National Center for Biotechnology Information (NCBI). In total, 11 foetoplacental arterial endothelial cells (AEC) from women with gestational diabetes mellitus (GDM) and 8 AEC specimens from normal pregnant women were enrolled in GSE103552 (platform: GPL6244 Affymetrix Human Gene 1.0 ST Array). In GSE104297 (GPL17303 Ion Torrent Proton), out of 28 AEC specimens, 14 were GDM and 14 were non‐GDM women Gene methylation profiling microarray data from 4 human GDM and 9 healthy pregnant controls in AEC samples in the NCBI GEO under accession number GSE106099 (platform: GPL13534 Illumina HumanMethylation450 BeadChip).

### 2.2 Data process

Raw gene expression profiles data were pre‐processed by R and Bioconductor packages. After background correction, logarithm transformation and normalization were conducted, differentially expressed genes (DEGs), differentially expressed miRNAs (DEMs) and differentially methylation probes (DMPs) were screened by using the *limma* package in R (version 3.6.0).^23^ After converting from probe level to gene level using the IlluminaHumanv4.db (version 1.26.0), DEGs, DEMs and DMPs were screened with *P* < 0.05 and | t |> 2 as the cut‐off criteria. DMPs located in the gene region were assigned to the corresponding genes, which were defined as differentially methylation genes (DMGs). To find out epigenetic alternations in GDM, jvenn online software (http://jvenn.toulouse.inra.fr/app/example.html) was adopted to identify overlapping genes from the DEG in GES103552 and DMGs in GSE106099. Subsequently, DEGs and potential targets of DEMs were overlapped to obtained low miRNA‐targeting up‐regulated genes and high miRNA‐targeting down‐regulated genes. Besides, hypomethylation–high‐expression genes and hypermethylation–low‐expression genes were summarized and obtained through overlapping aberrant methylated and expressed genes.

### 2.3 Prediction of miRNA Target genes and Construction of miRNA–mRNA Network

The target genes of DEMs were predicted by using miRWalk online database (http://zmf.umm.uni‐heidelberg.de/apps/zmf/mirwalk/index.html), which includes five different databases to predict miRNA target genes (miRanda, miRDB, Targetscan, RNA22 and miRWalk). Predicted genes which were fitted at least 3 databases were considered as the target gene of DEMs. After aligning DEMs and DEGs, we used the Cytoscape tool (v 3.7.1) to visualize the entire miRNA‐mRNA network.

### 2.4 Functional and pathway enrichment analysis

Gene ontology (GO) analysis and Kyoto Encyclopedia of Genes and Genomes (KEGG) pathway enrichment analysis were conducted for the hypomethylation‐high expression genes and hypermethylation–low‐expression genes selected by DAVID (Database for Annotation, Visualization and Integrated Discovery) (https://david.ncifcrf.gov/home.jsp). Besides, GO and Reactome pathways of hypomethylation‐high‐expression genes and hypomethylation‐high‐expression genes in the module were performed with the PANTHER classification system (http://geneontology.org) which uses many publicly available biological databases to identify interactions among the input gene list with *P* < 0.05 as the screening condition.

### 2.5 Protein‐protein Interaction (PPI) Network Construction and Module Analysis

The Search Tool for the Retrieval of Interacting Genes (STRING) online tool was used to construct a PPI network of hypomethylation‐high expression genes and hypermethylation–low‐expression genes, respectively. After PPI was visualized (combined score >0.7), modules within the PPI network was obtained by the Molecular Complex Detection (MCODE) in Cytoscape software. MCODE score >3 and the number of nodes >4. The functional enrichment analysis of the genes in individual modules was achieved by DAVID with a significance threshold of *P*<0.05. Hub genes were screened with connection degree >10.

### 2.6 Drug discovery in CMap

CMap (Connectivity Map) database (https://www.broadinstitute.org) is an open database that can be used to identify connections among small molecules which sharing a mechanism of chemicals, physiological processes and action, and then predict potential drugs in silicon.^24^ CMap analysis is used to predict potential small molecular compounds which can induce or reverse the altered expression of DEGs in cell lines. The link between the chemicals and query genes was measured via a connectivity score ranged from −1 to 1 and *P* < 0.05.

### 2.7 Molecular docking analysis

Molecular docking can more intuitively show and predict the interaction between compounds and target proteins encoded by miRNA‐associated aberrant methylation DEGs via Autodock (version 4.2.6). Protein crystal structures were downloaded from PDB (Protein Data Bank, https://www.wwpdb.org) and chemical structures were obtained from zinc15 online database (http://zinc.docking.org). First, the protein crystal structures were imported into Atutodock tools. Following the removal of irrelevant water molecules and ions, the addition of polar hydrogen atoms and assignment AD4 type, the proteins were prepared for docking. Compounds in the mol2 format were then imported into the software and protein‐ligand docking was run using a genetic algorithm with an optimized genetic algorithm.^25^

## 3 RESULTS

### 3.1 The microarray data information and identification of aberrantly methylated‐differentially expressed genes in GDM

The characteristics of the studies based on the GEO dataset are presented in Table S1. In GSE103552, a total of 5212 DEGs were identified in AEC samples from GDM, including 2095 up‐regulated genes and 3117 down‐regulated genes. Meanwhile, 16 high‐expressed miRNAs and 12 low‐expressed miRNAs were screened in miRNAs datasets of GSE104297. The detailed characteristics of the top 5 differentially expressed miRNAs and their corresponding target DEGs are shown in Table S2 (except has‐miR‐191‐3p owing to no targeting DEGs).

**FIGURE 1:**
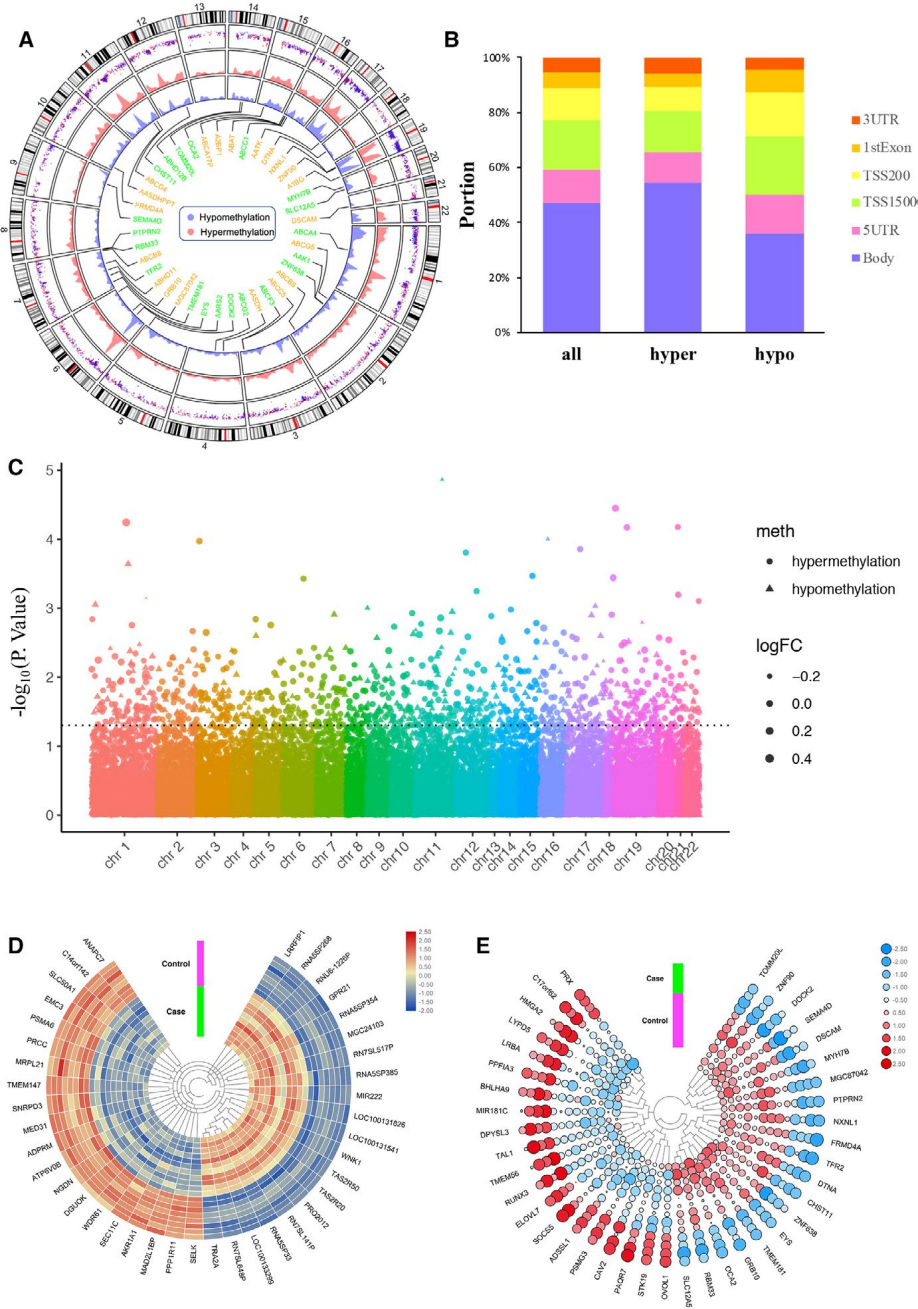
Differential DNA methylation distribution and hierarchical clustering heatmap of DEGs and DMGs. A, Circus plot of CpGs. Chromosomes are shown in a clockwise direction from 1 to 22 in the outermost circle. Chromosomes X, Y were excluded from analysis. Green‐ and orange‐labelled genes correspond to top 20 hypermethylated and hypomethylated genes, respectively. The two innermost circles represent the differential hypermethylation and hypomethylation frequencies in a 10 Mb sliding window across the genome. B, Bar plot of differentially methylated CpGs throughout each genomic region. C, Manhattan plot of epigenome‐wide association results showing ‐log10 (*P*‐value) regarding GDM. D, Top 40 DEGs (20 up‐regulated genes and 20 down‐regulated genes) of GSE106099. E, Top 40 DMEs (20 hypermethylation genes and 20 hypomethylation genes) of GSE103552. Red indicates that the expression of genes is relatively up‐regulated or the level of methylation is hypermethylated, blue indicated that the expression of genes is relatively down‐regulated or the level of methylation is hypomethylated

As for genes methylation microarray, 7387 hypomethylated CpG sites located within 4553 genes and 10010 hypermethylated CpG sites located within 5607 genes were found in GSE106099. All differentially methylated CpG sites from each autosomal chromosome are shown in the circus plot (Figure 1A). The distribution of differentially methylated CpG sites in six different genomic subregions is shown in Figure 1B. Additionally, differentially methylation genes (DMGs) are evenly distributed on autosomes (Figure 1C).

**FIGURE 2:**
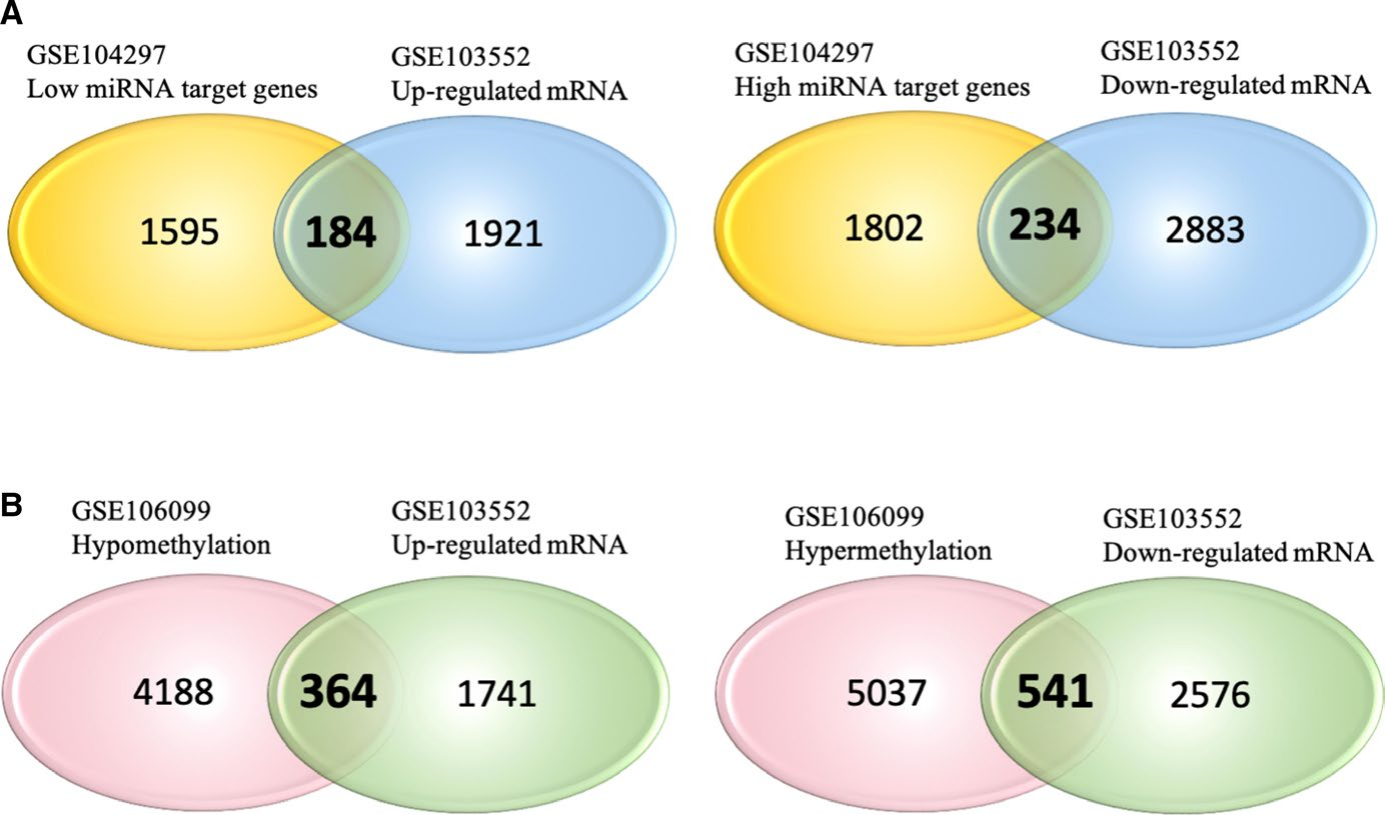
Identification of target genes of differentially expressed miRNAs and mRNA as well as aberrantly methylated‐differentially expressed genes between GDM and healthy samples. A, Target genes of differentially expressed miRNAs and mRNAs. B, Aberrantly methylated‐differentially expressed genes

Finally, 184 low miRNA‐targeting up‐regulated genes and 234 high miRNA‐targeting down‐regulated genes were identified though overlapping DEGs and target gene of DEMs, except has‐miR‐191‐3p, which has no overlapping targeting genes (Figure 2A). Moreover, 364 hypomethylation‐high expression genes and 541 hypermethylation–low‐expression genes via overlapping aberrant methylation and regulated genes were obtained (Figure 2B).

Furthermore, heatmaps of the top 40 DEGs and DMGs (20 up‐regulated and 20 down‐regulated genes as well as 20 hypermethylation and hypomethylation genes) are shown in Figure 1D and 1E, which suggested that they can be distinguished between GDM and normal.

### 3.2 DEGS associated with altered targeting miRNAs

#### 3.2.1 Up‐regulated genes and Low‐expression miRN

**FIGURE 3:**
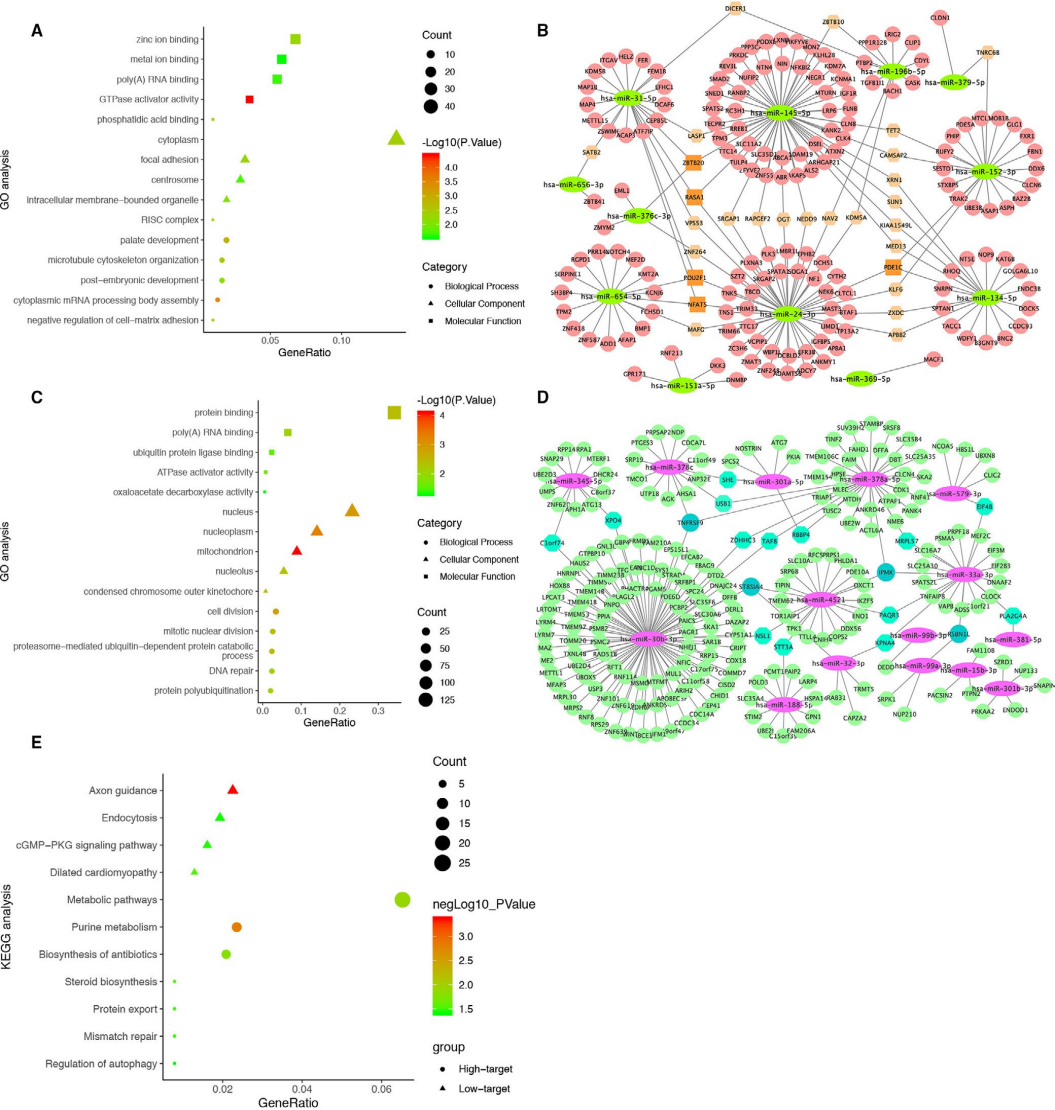
The visualized regulatory network and enrichment bubble graph for miRNA‐targeting DEGs. A, Enrichment bubble graph for up‐regulated genes. B, Regulatory network graph of 12 low‐expression miRNAs. C, Enrichment bubble graph for down‐regulated genes. D, Regulatory network graph of 15 high‐expression miRNAs (1 miRNA excluded). E, The KEGG enrichment bubble chart of Low‐Expression miRNA and Up‐Regulated genes and High‐Expression miRNA and Down‐Regulated Genes

Functional enrichment analysis identified 28 gene ontology (GO, including biological process, cellular component and molecular function) terms satisfying the thresholds of *P*‐value < 0.05, which were mainly associated with the regulation of transcription and metal ion binding (Table S3, Figure 3A). KEGG pathway enrichment analysis revealed that these genes were significantly enriched in pathways including Axon guidance, Dilated cardiomyopathy, cGMP‐PKG signalling pathway, and Endocytosis. In order to further identify important miRNA/mRNA regulated in GDM progression, the miRNA‐mRNA network was constructed (Figure 3B). ZBTB20, RASA1, POU2F1, NFAT5 and PDE1C were targeted by three miRNAs, and SATB2, DICER1, ZBTB10, TNRC6B, LASP1, VPS53, ZNF264, MAFG, SRGAP1, RAPGEF2, OGT, NEDD9, NAV2, KDM5A, TET2, CAMSAP2, XRN1, SUN1, KIAA1549L, MED13, KLF6, ZXDC and APBB2 were targeted by two miRNAs. The KEGG enrichment bubble chart of Low‐Expression miRNA and Up‐Regulated genes is shown in Figure 3E.

### 3.3 Down‐regulated genes and high‐expression miRNA

Enrichment analysis of high miRNA‐targeting down‐regulated genes suggested that 45 GO terms were identified with the thresholds of *P*‐value <0.05, which were mainly associated with cell division and protein binding (Table S3, Figure 3C). The most enriched KEGG analysis terms were Purine metabolism, Metabolic pathways, Biosynthesis of antibiotics, Steroid biosynthesis and Mismatch repair (Figure 3E). From the miRNA‐mRNA network, a total of 14 genes were regulated by two miRNAs and TNFRSF9, ST8SIA4, IPMK and RSBN1L were regulated by three miRNAs (Figure 3D). No overlapping has‐miR‐191‐3p significant target genes were predicted in miRWalk.

### 3.4 DEGS associated with altered DNA methylation

#### 3.4.1 High‐Expression and Hypomethylation Genes

**FIGURE 4:**
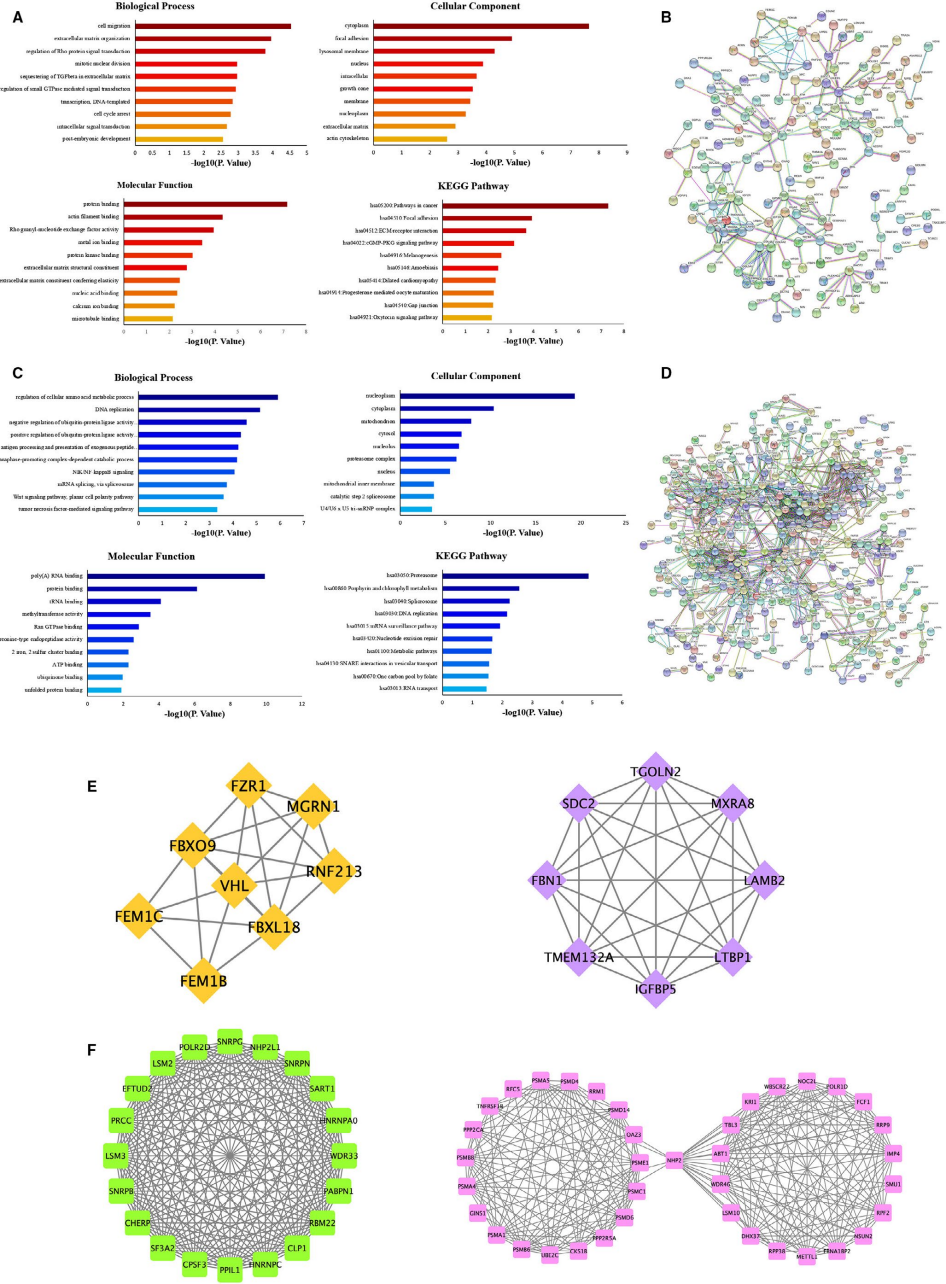
The protein‐protein interaction (PPI) network and enrichment bar graph for methylation‐related DEGs. A, The enrichment bar graph for hypomethylation–up‐regulated genes. B, PPI network of hypomethylation and high‐expression genes between GDM and healthy samples. C, The enrichment bar graph for hypermethylation–down‐regulated genes. D, PPI network of hypermethylation and low‐expression genes between GDM and healthy samples. E, Top two modules PPI network of hypomethylation–high‐expression genes. F, Top two modules PPI network of hypermethylation–low‐expression genes

Total 364 hypomethylation‐high‐expression genes were enriched in 116 GO enrichment terms with the thresholds of *P*‐value <0.05, such as signal transduction and cell migration (Table S4, Figure 4A). KEGG pathway analysis indicated enrichment of Pathways in cancer, Focal adhesion and ECM‐receptor interaction (Figure 4A). In total, 532 nodes and 1153 edges are shown in the PPI network (Figure 4B).

The top ten genes ranked by degree were identified as hub genes, included ABL1, COL4A2, FBN1, LAMB2, LTBP1, POLR2A, RHOT2, SDC2, TGOLN2 and VHL (Table S5). In these 10 hub genes, POLR2A is presented with the highest degree (degree = 31). Moreover, in order to study the important modules found in the PPI network, the top two significant modules were selected by MCODE plug‐in with 8.00 and 6.27 scores (Figure 4E), respectively, and the functional annotation of the genes involved in the modules was analysed using PANTHER classification system (http://geneontology.org). GO analysis showed that module 1 and module 2 were mainly related to TGFbeta in the extracellular matrix and protein polyubiquitination, respectively (data was not shown). Furthermore, Reactome pathway analysis enriched module 1 and module 2 genes in pathways of post‐translational protein phosphorylation and neddylation.

### 3.5 Low‐expression and Hypermethylation Genes

For hypermethylation–low‐expression genes, 117 GO terms were recognized with the thresholds of *P*‐value <0.05 in the DAVID database (Table S4, Figure 4C). KEGG pathway analysis identified enriched pathways of Proteasome, Porphyrin and chlorophyll metabolism, Spliceosome and DNA replication. In total, 359 nodes and 248 edges were shown in the PPI network (Figure 4D).

The nodes with the top 10 degrees were screened as hub genes, including the cluster of CPSF3, EFTUD2, HNRNPC, LSM2, NHP2L1, POLR2D, PPP2CA, RPP38, SNRPB, SNRPG and UBE2C. In these 11 hub genes, NHP2L1 and SNRPG presented with the highest degree (degree = 37) (Table S5).

Moreover, a total of 359 nodes were analysed using the plug‐in MCODE. The top 2 significant modules with 20.00 and 13.35 scores were selected (Figure 4F). Enrichment analysis of Modules 1 and 2 indicated that hypermethylation targeting down‐regulated genes participate in the termination of RNA polymerase II transcription and proteasomal ubiquitin‐independent protein catabolic process, respectively. Reactome pathway enrichment analysis revealed that modules 1 and 2 were significantly enriched in pathways, including mRNA Splicing and Regulation of ornithine decarboxylase.

### 3.6 DEGs associated with Both DNA Methylation and Aberrant miRNA

**FIGURE 5:**
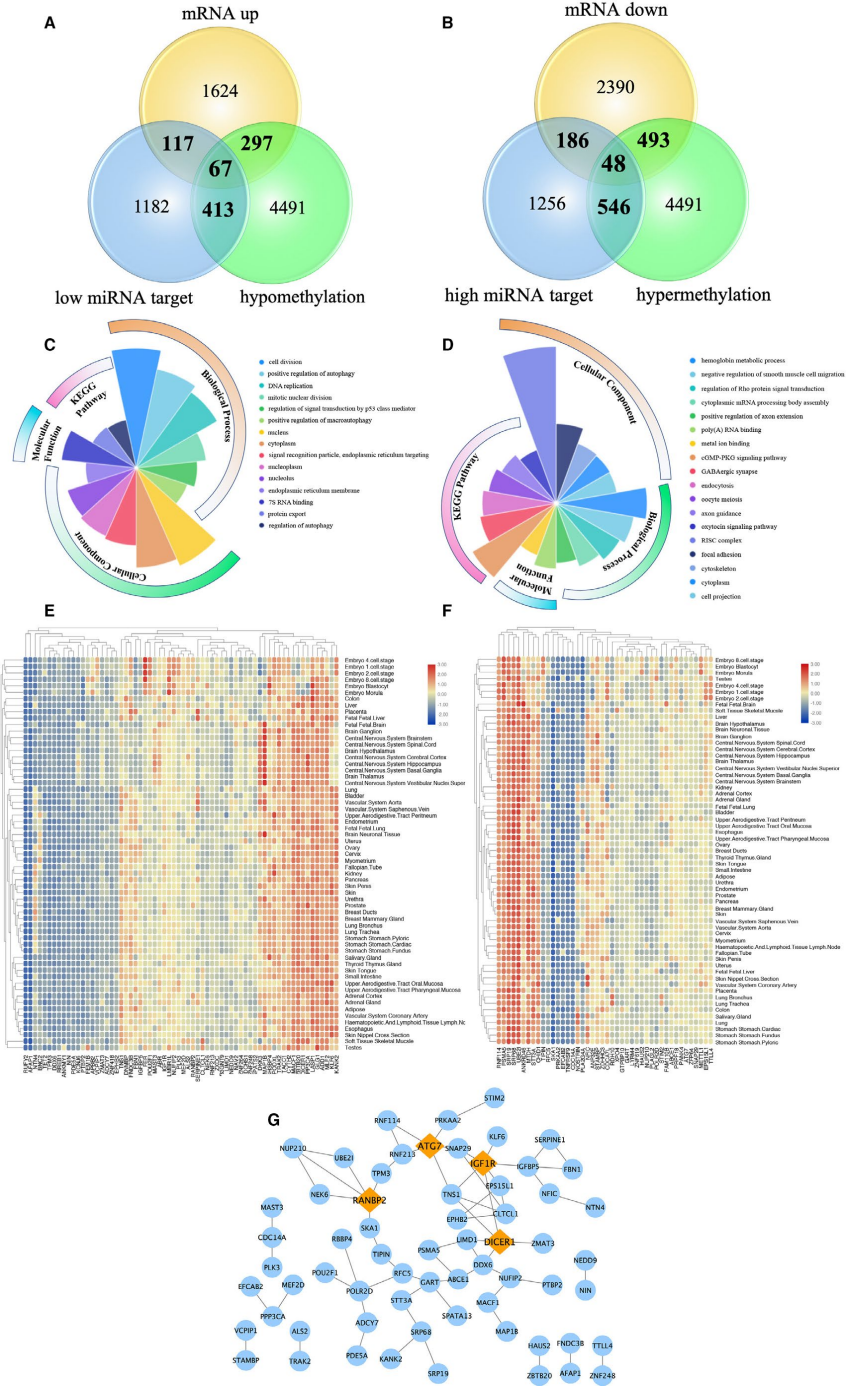
Details for all the overlapped genes. A and B, Venn graph for all the overlapped genes including up‐regulated 67 genes and 48 down‐regulated genes, respectively. C and D, Enrichment analysis of up‐regulated and down‐regulated genes, respectively. E, Heatmap of 67 up‐regulated genes expressed in different tissues. F, Heatmap of 48 down‐regulated genes expressed in different tissues. G, PPI network of all the overlapped genes including up‐regulated 67 genes and 48 down‐regulated genes

**FIGURE 6:**
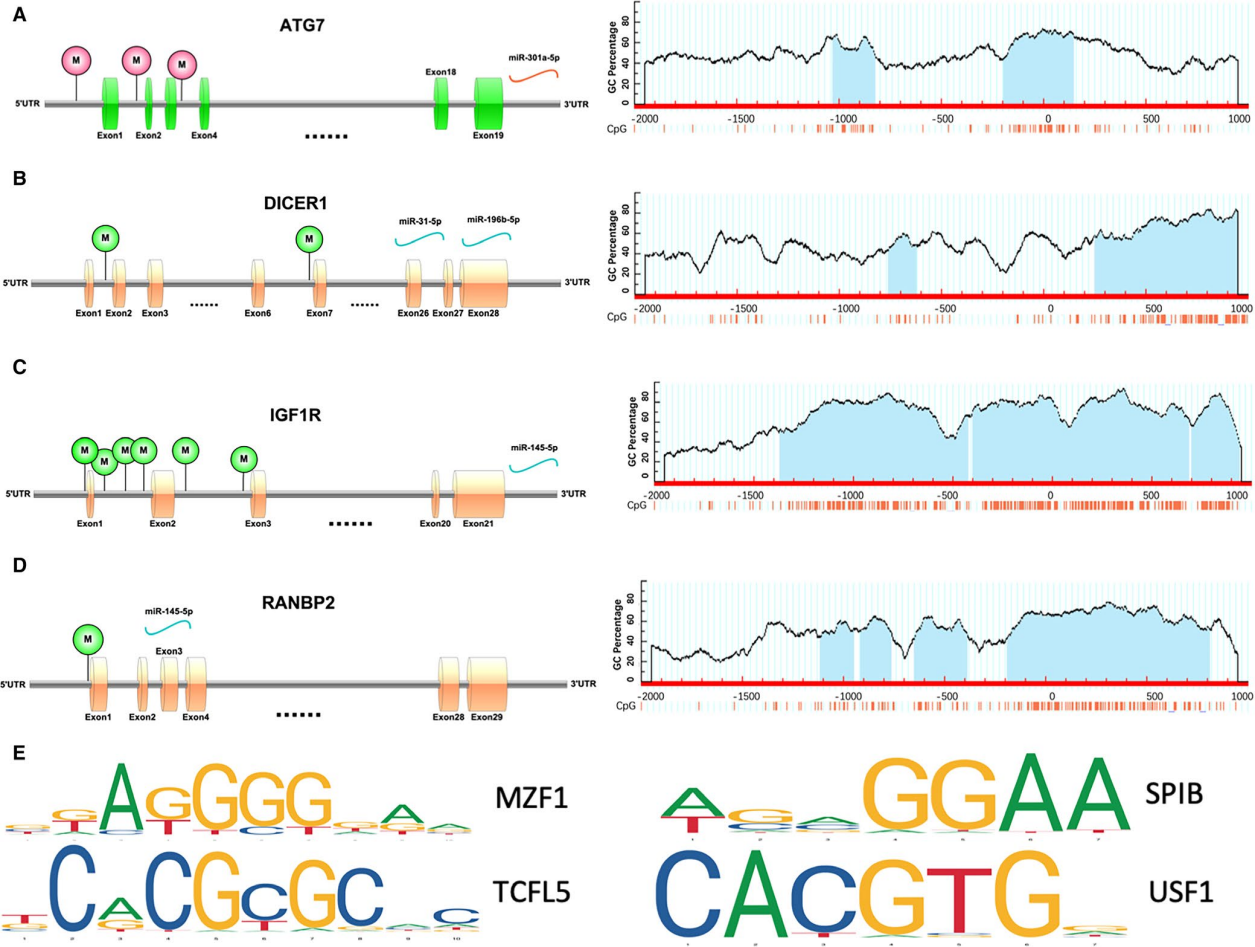
Details of the four screened genes. A–D, Epigenetic regulatory patterns of both miRNA and DNA methylation regulating gene expression as well as CpG islands prediction analysis of 4 screened genes. E, Representative predicted transcription factors of target IGF1R CpG islands identified to be enriched in DMRs by JASPAR database

Interestingly, several DEGs were regulated by both aberrant alternations of DNA methylation and miRNA, which might demonstrate more vital and valuable function underlying GDM. Sixty‐seven genes such as ABCE1, ANKRD46, ANP32E and ATG7 were up‐regulated under the modulation of both hypomethylation and decreased miRNA (Figure 5A). Simultaneously, forty‐eight genes, including SPATS2, SERPINE1, TACC1, ADD1 and NEK6 were down‐regulated under modulation of both hypermethylation and increased miRNA (Figure 5B). The DNA methylation site and its relation to CpG island, as well as the specific regulatory miRNA and binding site, are summarized in Table S6. Moreover, the results of functional enrichment analysis of up‐ or down‐regulated genes in GDM are shown in Figure 5C‐D, respectively. To clearly elucidate the more in‐depth insights into up‐ and down‐regulated gene expression across the entire human genome especially in all embryo cell stage and placenta tissues, MERAV online database (http://merav.wi.mit.edu) was used and established the representative expression heatmap of overlapped DEGs (67 up‐regulated and 48 down‐regulated genes) (Figure 5F and Figure 5G). Among the up‐regulated 66 genes (ACAP3 was not found in the MERAV database), SERPINE1, FBN1 and DICER1 have the highest expression level in the placenta. Among the down‐regulated 44 genes (C11orf49, C15orf39, C19orf47 and RBBP4 were not identified in MERAV database), SRP19, SRP68 and UBE2I have the highest expression level in placenta. The 115 genes, including 67 low miRNA‐targeting up‐regulated hypomethylation genes and 48 high miRNA‐targeting down‐regulated hypermethylation genes, were submitted to the CMap online tool to predict latent drugs in the therapy for GDM depending on the expression alteration. By ranking the connectivity score in descending order, the top 4 chemicals were identified as being potential treatment options for GDM (Table 1). Moreover, a protein‐protein interaction (PPI) network for all the overlapped aberrant expressed genes including up‐regulated 67 genes and 48 down‐regulated genes were constructed (Figure 5C). Four hub genes from the PPI (Figure 5C) were screened for further analysis, including three genes with up‐regulated expression levels under both hypomethylation and low miRNA regulation RANBP2, DICER1 and IGF1R, as well as ATG7 with down‐regulated expression level under both hypermethylation and high miRNA regulation. The representative epigenetic regulatory pattern of both miRNA and DNA methylation regulating expression on 4 selected genes are shown in Figure 6A‐D. Next, a CpG islands prediction analysis was conducted (Figure 6A‐D). RANBP2 and IGF1R have 4 and 3 predicted CpG islands, respectively. Both DICER1 and ATG7 have 2 predicted CpG islands. Furthermore, representative 4 CpG island binding transcription factors in the IGF1R promoter region were identified to be enriched in DMRs by CisGenome Browser software (Figure 6E). Moreover, to ascertain whether the three chemicals directly bind to the proteins encoded by the four genes, a protein‐ligand docking analysis was performed. The docking results are presented in *Supplementary Figure*. However, further experiments are required to verify these associations.

**TABLE 1:** Ten chemicals were predicted as putative therapeutic agents for GDM

CMap name	Chemical formula	Mean	n	Enrichment	*P*
trichostatin A	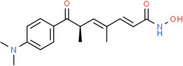	0.521	182	0.59	<0.00001
LY‐294002	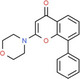	0.528	61	0.547	<0.00001
sirolimus	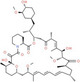	0.478	44	0.475	<0.00001
vorinostat	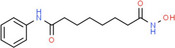	0.578	12	0.69	0.00002
puromycin	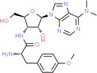	0.698	4	0.917	0.00004
alvespimycin	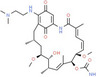	0.485	12	0.63	0.00008
helveticoside	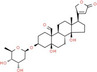	−0.242	6	−0.807	0.00014
ceforanide	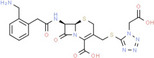	−0.569	4	−0.902	0.00016
valproic acid	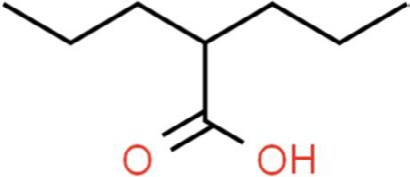	0.267	57	0.271	0.00032
thioridazine	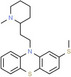	0.389	20	0.45	0.00038

## 4 DISCUSSION

Changes in the promoter DNA methylations and miRNA expression play vital roles in pregnancy‐related diseases by up‐ or down‐regulating gene expression.^26^, ^27^ DNA methylation pattern and miRNA expression alterations can serve as useful biomarkers to distinguish GDM from normal samples,^27^, ^28^ which would be used for clinical diagnosis, assessment of treatment and diagnosis.^29^ In the current study, data of miRNAs microarrays (GSE104297), DNA methylation microarrays (GSE106099) and mRNA microarrays (GSE103552) were systematically analysed which compare the differential profiling between AEC samples from GDM and controls. Core genes and pathways have been enriched to identify key events in epigenetic alternation regulated by microRNA and DNA methylation.

Until now, 184 low miRNA targeted up‐regulated genes through overlapping strategies of DEGs and targets of DEMs were finally identified. DAVID analysis showed that these 184 genes are mostly enriched in biological processes in GDM remind us of the probable involvement of embryo growth in GDM. The GO analysis showed that its molecular function was obviously related to metal ion binding function and energy metabolism suggested that the metal levels may be related to increased GDM risk.^30^ KEGG analysis revealed pathways including axon guidance, dilated cardiomyopathy, cGMP‐PKG signalling pathway and endocytosis. A cGMP‐PKG signalling pathway is a pathway that may regulate Ca_v_13 channels and contribute to regulating insulin secretion.^31^ Moreover, bradykinin can enhance insulin action by sGC‐cGMP‐PKG signalling pathway up‐regulated MKP‐5, inhibiting negative feedback of JNK and ERK.^32^ In addition, five up‐regulated genes, including ZBTB20, RASA1, POU2F1, NFAT5 and PDE1C were targeted by three miRNAs and 23 genes including APBB2, CAMSAP2 and DICER1 were targeted by two miRNAs, but their functions in GDM were poorly understood.

A total of 234 high miRNAs targeted down‐regulated genes were given through overlapping DEG and DEM targets, which suggested that these genes can affect the transcription and replication of cells from cellular component analysis. Numbers of studies have shown that metabolic pathway and steroid biosynthesis from KEGG analysis play an important role in the genesis and growth of gestational diabetes mellitus.^33^, ^34^ Notably, overall 18 genes were controlled by more than one miRNAs, which may be the intersection of multiple miRNA regulation. Previous research has shown that has‐miR‐30b‐3p had abundant target genes, such as PDE6D, POLR2D, PAICS and GART, associated with purine metabolism. A study found that purine metabolites and tryptophan were consistently up‐regulated in the urinary metabolome of GDM patients.^35^ Therefore, the value of miR‐30b‐3p in GDM should be explained carefully and further molecular research is needed to clarify.

The data showed that the 364 genes with low methylation and high expression were obtained through enrichment in cGMP‐PKG signalling pathway, ECM‐receptor interaction and focal adhesion, and melanogenesis. Therefore, hypomethylation‐induced imbalance of ECM‐receptor interaction and cGMP‐PKG signalling pathway may be the cause of GDM. The PPI network of low‐methylated and highly expressed genes outlines their functional connections, with the top 10 central genes among them such as ABL1, COL4A2, FBN1 and LAMB2 were also selected. In addition, a module analysis of the PPI network for low methylation and high expression genes showed that sequestering of TGF‐β in the extracellular matrix, protein polyubiquitination, post‐translational protein phosphorylation and neddylation, which showed that the most apparent changes seem to be the metabolisms of various protein, are disorganized, inevitably alters.

For 541 low‐methylation and low‐expression genes through overlapping hypermethylation and down‐regulation in GDM, GO and KEGG analysis showed displayed enrichment in cellular amino acid metabolic process and proteasome, respectively. From the PPI network, the top 10 hub genes of hypermethylation–low‐expression genes were screened and two core modules possessed functions, including sequestering of T mRNA splicing, rRNA processing, spliceosome and proteasome which may participate in GDM development. mRNA splicing, rRNA processing and spliceosome are whole key cellular processes in the process of post‐transcriptional modification and regulation, which may be confused during pregnancy.^36^

It is very interesting that epigenetic alternations of miRNA and DNA methylation may work in concert to identify aberrant expression of some genes in GDM. Sixty‐seven genes such as DICER1, IGF1R, SERPINE1, FBN1 and RANBP2 were raised due to the regulation of both decreased miRNAs, while under the modulation of both increased miRNA, forty‐eight genes including ATG7, SERPINE1 and FBN1 were down‐regulated. For 67 low miRNA‐targeting up‐regulated hypomethylation genes, GO analysis identified enrichment in haemoglobin metabolic processes, negative regulation of smooth muscle cell migration, regulation of Rho protein signal transduction, RISC complex and focal adhesion. Moreover, these genes are also involved in the cGMP‐PKG signalling pathway, GABAergic synapse, endocytosis and oocyte meiosis signalling pathways. The three high expressions of these genes (SERPINE1, FBN1 and DICER1) in the placenta suggest that they are involved in pregnancy function. For 48 high miRNA‐targeting down‐regulated hypermethylation genes, GO analysis identified enrichment in cell division, positive regulation of autophagy, DNA replication and 7S RNA binding. But no significant results were retrieved from KEGG pathway analysis. Among these genes, SRP19, SRP68 and UBE2I have the highest expression level in the placenta, which were related to the gestation process.

Since there are no effective drugs for gestational diabetes mellitus, the online database was used to aid the prediction of some drugs. At present, CMap is a tool of practical value for exploring new drugs and reusing existing drugs, and its effectiveness has been confirmed by many studies.^37^ From the CMap database, 10 chemicals, including trichostatin A, LY‐294002 and sirolimus were identified and may have significant potential therapeutic effects on gestational diabetes mellitus. Trichostatin A is an epigenetic modifier and may cause maternal‐foetal immune tolerance and reduce embryonic reabsorption in miscarriage prone mice,^38^ but more experimental studies are necessary in order to validate the therapeutic effects of these potential drugs on GDM. After constructing the PPI network of all overlapped genes illustrated, the top 4 hub genes appeared to be IGF1R, DICER1, RANBP2 and ATG7. IGF1R, one of the members of the insulin/IGF system, was mostly expressed in foetal and cancer tissues ^39^ and three CpG islands are predicted in the promoter region of IGF1R which may combine large list of transcript factors including MZF1, TCFL5, USF1, ETS1 and SPIB. However, the high level of IGF1R transcript in foetal heart and liver of pregnancy diabetes rats relative to controls was augmented.^40^ DICER1 contains two CpG islands in its promoter region and is an RNaseIII endonuclease that plays an important role in the process of processing pre‐miRNA into active mature miRNA and correlated with various tumours.^41^ Rahimi et al ^42^ indicated that the expression levels of Drosha, DGCR8 and Dicer were higher in pregnant women and GDM patients than in the control group, which suggested that these three genes might be involved and played vital roles in the pathogenesis of GDM. The Ranbp2 is a vital, large, mosaic, pleiotropic nucleoporin and localized at the cytoplasmic peripheral side of the nuclear pore complex, which commands proteostasis of selective substrates and the nuclear‐cytoplasmic trafficking in a cell‐type dependent manner.^43^, ^44^ In cancer cells, IGF‐1R first binds to the dynactin subunit p150 (glued), which transports the receptor to the nuclear pore complex where it co‐localizes with importin‐β and then binds to Ranbp2.^45^ However, Ranbp2 has never been linked directly to GDM, with only some preliminary explorations in the embryo.^46^ ATG7, as an important autophagy‐related protein, is involved in activating ubiquitin‐like protein (UBL), which is essential for the formation of autophagosome in the recognized pathway.^47^ Although the role of autophagy on GDM was controversial, autophagy was activated in GDM placentas^48^ and the birth weight of foetuses was significantly decreased in labyrinth layer‐specific ATG7 knockout mouse models.^49^

There are some deficiencies in our survey. Due to data availability, this study did not analyse the association of clinical data such as clinical parameters and prognosis with epigenetic changes. In addition, the effects of abnormal methylation and expression In addition, the effects of abnormal methylation and expression of miRNAs on gene expression were not validated in experiments. Thus, further evaluations in clinical trials are required to validate these genes.

## 5 CONCLUSION

This study indicated a series of aberrantly methylated‐differentially expressed genes that are associated with epigenetic alternations of miRNAs and DNA methylation in GDM. 184 low miRNA‐targeting up‐regulated genes and 234 high miRNA‐targeting down‐regulated genes were obtained by overlapping DEGs and targets of DEMs, which were enriched in the cGMP‐PKG signalling pathway and Purine metabolism, respectively. Moreover, 364 hypomethylation‐high expression genes by overlapping hypomethylation and up‐regulated genes, and 541 hypermethylation–low‐expression genes via overlapping hypermethylation and down‐regulated genes, were related to ECM‐receptor interaction and proteasome. Interestingly, 67 genes were up‐regulated by the modulation of both decreased miRNA and hypomethylation, while 48 genes were down‐regulated under the modulation of both increased miRNA and hypermethylation. Ten chemicals were identified as putative therapeutic agents for GDM. Moreover, from these genes, ATG7, DICER1, IGF1R and RANBP2 may play an important role in the genesis and growth of gestational diabetes mellitus and might serve as aberrantly methylation‐based or expression of miRNA‐targeting biomarkers for precise diagnosis and treatment of GDM in the future.

## CONFLICT OF INTEREST

The authors declare that they have no conflict of interests.

## AUTHORS’ CONTRIBUTION

ZWQ and SYP were responsible for the statistical analysis and wrote the manuscript. LJW, ZZF, LM, FXP, ZQX and XJH contributed to review and revision of the manuscript. LSQ, ZWJ, ZMH and DJ were responsible for the study design. All authors read and approved the final manuscript.

## ETHICS APPROVAL AND CONSENT TO PARTICIPATE

Not applicable.

## CONSENT FOR PUBLICATION

Not applicable.

## Supporting information

Fig S1

Table S1‐S6

## Data Availability

The authors declare that the data supporting the findings of this study are available within the article.
