# Supplementary figures and images for: Epigenetic alternations of microRNAs and DNA methylation contribute to gestational diabetes mellitus
Source: J Cell Mol Med. 2020 Oct 21;24(23):13899–912. doi: 10.1111/jcmm.15984 (PMC7753873; doi:10.1111/jcmm.15984)

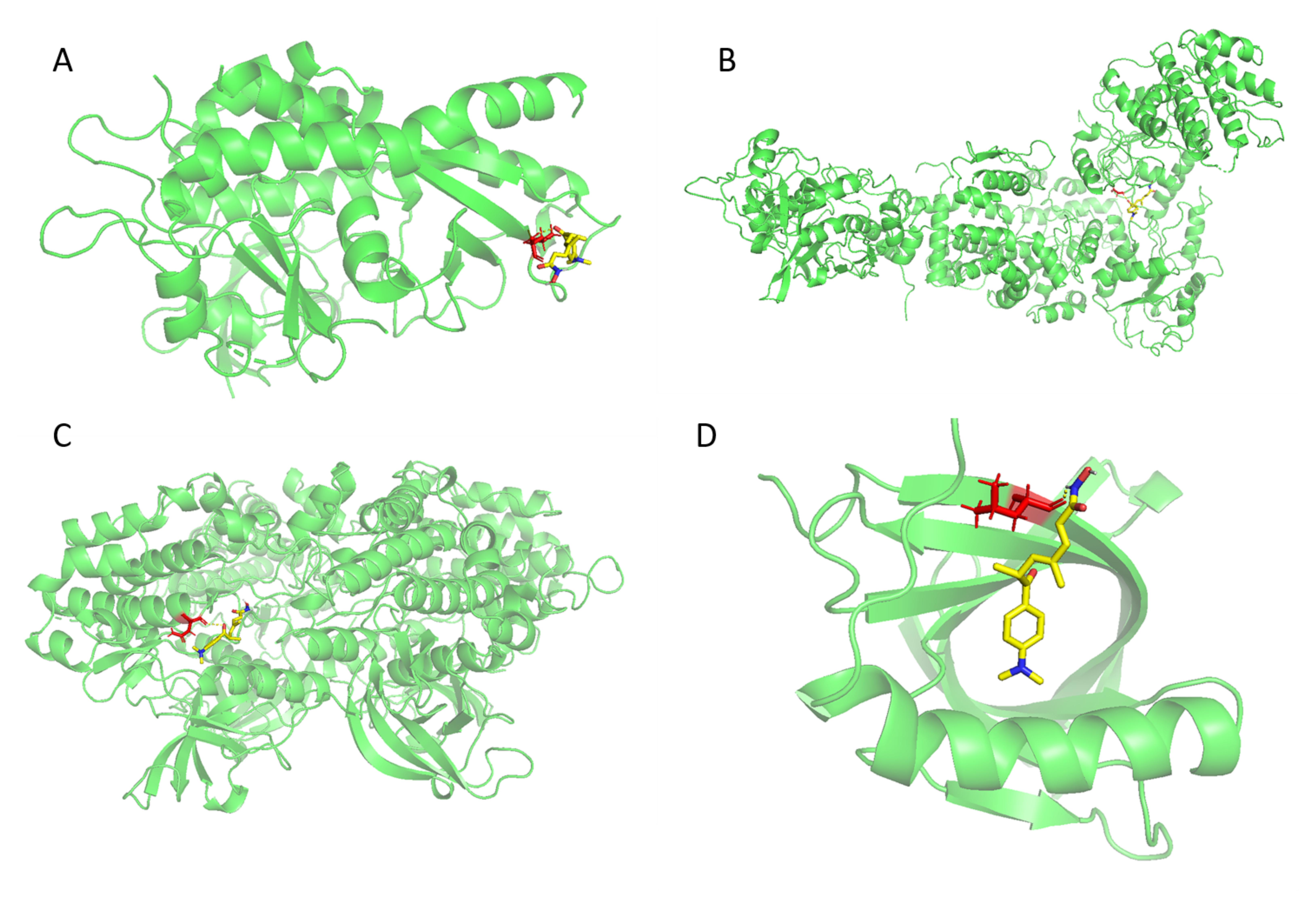

Supplement: Supplementary file 1 — Fig S1 [file JCMM-24-13899-s001.jpg]
