# Supplementary material for: Epigenetic alternations of microRNAs and DNA methylation contribute to gestational diabetes mellitus
Source: J Cell Mol Med. 2020 Oct 21;24(23):13899–912. doi: 10.1111/jcmm.15984 (PMC7753873; doi:10.1111/jcmm.15984)
Supplement: Supplementary file 2 — Table S1‐S6 [file JCMM-24-13899-s002.docx]

**Epigenetic alternations of microRNAs and DNA methylation contribute to gestational diabetes mellitus**

Weiqiang Zhu^1,2^††, Yupei Shen^2^††, Junwei Liu^2^, Xiaoping Fei^4^, Zhaofeng Zhang^2^, Min Li^2^, Xiaohong Chen^5^, Jianhua Xu^2^, Qianxi Zhu^2^, Weijin Zhou^2^, Meihua Zhang^1^*, Shangqing Liu^3^*, Jing Du^2^*

^1^ Key Laboratory of Birth Regulation and Control Technology of National Health Commission of China, Shandong Provincial Maternal and Child Health Care Hospital Affiliated to Shandong University, Jinan 250001, China.

^2^ NHC Key Lab. of Reproduction Regulation (Shanghai Institute of Planned Parenthood Research), Pharmacy School, Fudan University, Shanghai 200032, China

^3^ North Sichuan Medical College

^4^ The First people's Hospital of Kunshan

^5^ Department of Obstetrics and Gynecology, Shanghai Pudong New Area Health Care Hospital For Women & Children, Shanghai 201206, China

††The first two authors are similar in author order.

Supplementary Table 1. Datasets used in finally quantitative synthesis and integrated analysis.

| **Dataset** | **Author** | **Platform** | **Samples(N : P)^a^** | **Expression type** | **Year** |
| --- | --- | --- | --- | --- | --- |
| GSE106099 | Cvitic S, et al | GPL13534 Illumina HumanMethylation450 BeadChip | 4 : 9 | Methylation profiling | 2018 |
| GSE103552 | Cvitic S, et al | GPL6244 Affymetrix Human Gene 1.0 ST Array | 11 : 8 | mRNA expression | 2018 |
| GSE104297 | Hiden U, et.al | GPL17303 Ion Torrent Proton | 14 : 14 | MIRNA expressioin | 2019 |

Note: ^a^N, nomal people; P, patients

Supplementary Table 2. The detailed characteristics of the top 5 differentially expression miRNAs.

|  | **ID*** | **sequence** | **chr** | **target genes** |
| --- | --- | --- | --- | --- |
| ***Down-regulated*** |  |  |  |  |
| hsa-miR-134-5p | MI0000474 | UGUGACUGGUUGACCAGAGGGG | 14q32.31 | APBB2,B3GNT9,CCDC93,DOCK5,FNDC3B,GOLGA6L10,KIAA1549L,NOP9,PDE1C,RHOQ |
| hsa-miR-145-5p | MI0000461 | GUCCAGUUUUCCCAGGAAUCCCU | 5q32 | ABR,ADAM19,ARHGAP21,CLN8,DSEL,IGF1R,KANK2,KCNMA1,KDM7A,KIAA1549L |
| hsa-miR-656-3p | MI0003678 | AAUAUUAUACAGUCAACCUCU | 14q32.31 | KLF6,ZBTB41,SATB2 |
| hsa-miR-376c-3p | MI0000776 | AACAUAGAGGAAAUUCCACGU | 14q32.31 | ZBTB20,ZMYM2, EML1 |
| hsa-miR-31-5p | MI0000089 | AGGCAAGAUGCUGGCAUAGCU | 9p21.3 | APBB2,ATF7IP,CEP85L,DICER1,EFHC1,FEM1B,FER,HELZ,KDM5B,KIAA1549L |
| ***Up-regulated*** |  |  |  |  |
| hsa-miR-378a-5p | MI0000786 | CUCCUGACUCCAGGUCCUGUGU | 5q32 | DBT,DFFA,FAIM,HPSE,IPMK,MLEC,MRPL57,NME6,RBBP4,SKA2 |
| hsa-miR-4521 | MI0016887 | GCUAAGGAAGUCCUGUGCUCAG | 17p13.1 | CNIH4,COPS2,DDX56,ENO1,IPMK,NSL1,PDE10A,PHLDA1,PRPS1,SLC10A7 |
| hsa-miR-32-3p | MI0000090 | CAAUUUAGUGUGUGUGAUAUUU | 9q31.3 | KPNA4,RAB31,ST8SIA4,TRMT5,CAPZA2 |
| hsa-miR-99a-3p | MI0000101 | CAAGCUCGCUUCUAUGGGUCUG | 21q21.1 | SRPK1,NUP210,RSBN1L |
| hsa-miR-188-5p | MI0000230 | CAUCCCUUGCAUGGUGGAGGG | Xp11.23 | C15orf39,FAM206A,GPN1,POLD3,SLC35A4,UBE2I,PAIP2,HSPA14,LARP4,PCMT1 |

*miRBase ID

Chr chromosome band. If there are more than ten target genes, only ten are listed in table.



Supplementary Table 3. Gene ontology and KEGG pathway analysis of DEGs targeted by altered miRNAs between GDM and healthy samples.

| **Category** | **Term** | **Count** | **Precent (%)** | **P value** |
| --- | --- | --- | --- | --- |
| ***Low miRNA targeting up-regulated genes*** | |  |  |  |
| Biological Process | cytoplasmic mRNA processing body assembly | 4 | 1.29 | 3.25E-04 |
| Biological Process | palate development | 6 | 1.93 | 0.001293217 |
| Biological Process | microtubule cytoskeleton organization | 5 | 1.61 | 0.002994961 |
| Biological Process | negative regulation of cell-matrix adhesion | 3 | 0.97 | 0.005818107 |
| Biological Process | post-embryonic development | 5 | 1.61 | 0.007950475 |
| Cellular Component | cytoplasm | 43 | 13.85 | 0.004054644 |
| Cellular Component | focal adhesion | 10 | 3.22 | 0.005325293 |
| Cellular Component | RISC complex | 3 | 0.97 | 0.006265062 |
| Cellular Component | intracellular membrane-bounded organelle | 6 | 1.93 | 0.008684909 |
| Cellular Component | centrosome | 9 | 2.90 | 0.019854948 |
| Molecular Function | GTPase activator activity | 11 | 3.54 | 3.72E-05 |
| Molecular Function | phosphatidic acid binding | 3 | 0.97 | 0.004707126 |
| Molecular Function | zinc ion binding | 21 | 6.76 | 0.005396829 |
| Molecular Function | poly(A) RNA binding | 17 | 5.47 | 0.023302707 |
| Molecular Function | metal ion binding | 18 | 5.80 | 0.028791087 |
| KEGG Pathway | axon guidance | 7 | 2.25 | 4.28E-04 |
| KEGG Pathway | dilated cardiomyopathy | 4 | 1.29 | 0.029256513 |
| KEGG Pathway | cGMP-PKG signaling pathway | 5 | 1.61 | 0.03625721 |
| KEGG Pathway | endocytosis | 6 | 1.93 | 0.037653772 |
| ***High miRNA targeting down-regulated genes*** | |  |  |  |
| Biological Process | cell division | 13 | 3.39 | 0.001324114 |
| Biological Process | mitotic nuclear division | 10 | 2.61 | 0.003629382 |
| Biological Process | proteasome-mediated ubiquitin-dependent protein catabolic process | 9 | 2.35 | 0.003638438 |
| Biological Process | protein polyubiquitination | 8 | 2.09 | 0.007685121 |
| Biological Process | DNA repair | 9 | 2.35 | 0.008584905 |
| Cellular Component | mitochondrion | 34 | 8.87 | 8.06E-05 |
| Cellular Component | nucleoplasm | 54 | 14.09 | 5.43E-04 |
| Cellular Component | nucleus | 89 | 23.22 | 0.001104428 |
| Cellular Component | condensed chromosome outer kinetochore | 3 | 0.78 | 0.003976032 |
| Cellular Component | nucleolus | 21 | 5.48 | 0.004383869 |
| Molecular Function | protein binding | 131 | 34.18 | 0.003855282 |
| Molecular Function | poly(A) RNA binding | 25 | 6.52 | 0.008000801 |
| Molecular Function | ATPase activator activity | 3 | 0.78 | 0.021042832 |
| Molecular Function | ubiquitin protein ligase binding | 9 | 2.35 | 0.02870726 |
| Molecular Function | oxaloacetate decarboxylase activity | 2 | 0.52 | 0.049299892 |
| KEGG Pathway | purine metabolism | 9 | 2.35 | 0.001517637 |
| KEGG Pathway | metabolic pathways | 25 | 6.52 | 0.012490932 |
| KEGG Pathway | biosynthesis of antibiotics | 8 | 2.09 | 0.016413883 |
| KEGG Pathway | steroid biosynthesis | 3 | 0.78 | 0.025367216 |
| KEGG Pathway | mismatch repair | 3 | 0.78 | 0.032974426 |

Supplementary Table 4. Gene ontology and KEGG pathway analysis of DEGs associated with aberrant DNA methylation between GDM and healthy samples.

| **Category** | **Term** | **Count** | **Precent (%)** | ***P* value** |
| --- | --- | --- | --- | --- |
| ***Hypomethylation and up-regulated genes*** | | | | |
| Biological Process | cell migration | 14 | 2.53 | 2.91196E-05 |
| Biological Process | extracellular matrix organization | 14 | 2.53 | 0.000111499 |
| Biological Process | regulation of Rho protein signal transduction | 9 | 1.63 | 0.000163295 |
| Biological Process | mitotic nuclear division | 14 | 2.53 | 0.001065545 |
| Biological Process | sequestering of TGFbeta in extracellular matrix | 3 | 0.54 | 0.001092521 |
| Cellular Component | cytoplasm | 144 | 26.06 | 2.21836E-08 |
| Cellular Component | focal adhesion | 22 | 3.98 | 1.24599E-05 |
| Cellular Component | lysosomal membrane | 17 | 3.08 | 5.09003E-05 |
| Cellular Component | nucleus | 132 | 23.89 | 0.000130559 |
| Cellular Component | intracellular | 44 | 7.96 | 0.000220446 |
| Molecular Function | protein binding | 216 | 39.09 | 6.65946E-08 |
| Molecular Function | actin filament binding | 12 | 2.17 | 4.69329E-05 |
| Molecular Function | Rho guanyl-nucleotide exchange factor activity | 9 | 1.63 | 0.000110107 |
| Molecular Function | metal ion binding | 62 | 11.22 | 0.000357706 |
| Molecular Function | protein kinase binding | 18 | 3.26 | 0.000975524 |
| KEGG Pathway | pathways in cancer | 26 | 4.71 | 4.95819E-08 |
| KEGG Pathway | focal adhesion | 14 | 2.53 | 0.000117697 |
| KEGG Pathway | ECM-receptor interaction | 9 | 1.63 | 0.000207632 |
| KEGG Pathway | cGMP-PKG signaling pathway | 11 | 1.99 | 0.000724179 |
| KEGG Pathway | melanogenesis | 8 | 1.45 | 0.002613345 |
| ***Hypermethylation and down-regulated genes*** | | | | |
| Biological Process | regulation of cellular amino acid metabolic process | 11 | 1.31 | 1.22E-06 |
| Biological Process | DNA replication | 17 | 2.03 | 7.37E-06 |
| Biological Process | negative regulation of ubiquitin-protein ligase activity involved in mitotic cell cycle | 11 | 1.31 | 2.68E-05 |
| Biological Process | positive regulation of ubiquitin-protein ligase activity involved in regulation  of mitotic cell cycle transition | 11 | 1.31 | 4.90E-05 |
| Biological Process | antigen processing and presentation of exogenous peptide antigen via MHC  class I, TAP-dependent | 10 | 1.19 | 6.04E-05 |
| Cellular Component | nucleoplasm | 156 | 18.63 | 4.65E-20 |
| Cellular Component | cytoplasm | 210 | 25.07 | 4.37E-11 |
| Cellular Component | mitochondrion | 73 | 8.72 | 1.36E-08 |
| Cellular Component | cytosol | 137 | 16.36 | 1.67E-07 |
| Cellular Component | nucleolus | 51 | 6.09 | 3.07E-07 |
| Molecular Function | poly(A) RNA binding | 72 | 8.60 | 1.16E-10 |
| Molecular Function | protein binding | 300 | 35.82 | 7.51E-07 |
| Molecular Function | tRNA binding | 9 | 1.07 | 8.15E-05 |
| Molecular Function | methyltransferase activity | 11 | 1.31 | 3.09E-04 |
| Molecular Function | Ran GTPase binding | 6 | 0.72 | 0.001375527 |
| KEGG Pathway | proteasome | 10 | 1.19 | 1.36E-05 |
| KEGG Pathway | porphyrin and chlorophyll metabolism | 7 | 0.84 | 0.002788214 |
| KEGG Pathway | spliceosome | 12 | 1.43 | 0.005628739 |
| KEGG Pathway | DNA replication | 6 | 0.72 | 0.007074272 |
| KEGG Pathway | mRNA surveillance pathway | 9 | 1.07 | 0.012206961 |

Supplementary Table 5. Hub genes with the top 10 degrees of both high expression and low expression genes.

| **Gene** | **Gene Description** | **Degree** |
| --- | --- | --- |
| ***Hypomethylation and High-Expression hub Genes*** | |  |
| POLR2A | RNA polymerase II subunit A | 14 |
| TGOLN2 | trans-golgi network protein 2 | 12 |
| SDC2 | syndecan 2 | 11 |
| ABL1 | ABL proto-oncogene 1, non-receptor tyrosine kinase | 10 |
| FBN1 | fibrillin 1 | 10 |
| LAMB2 | laminin subunit beta 2 | 10 |
| VHL | von Hippel-Lindau tumor suppressor | 10 |
| RHOT2 | ras homolog family member T2 | 9 |
| COL4A2 | collagen type IV alpha 2 chain | 8 |
| LTBP1 | latent transforming growth factor beta binding protein 1 | 8 |
| ***Hypermethylation and Low-Expression hub Genes*** | |  |
| SNRPG | small nuclear ribonucleoprotein polypeptide G | 37 |
| NHP2L1 | U4/U6 small nuclear ribonucleoprotein | 37 |
| POLR2D | RNA polymerase II subunit D | 35 |
| SNRPB | small nuclear ribonucleoprotein polypeptides B and B1 | 32 |
| PPP2CA | protein phosphatase 2 catalytic subunit alpha | 29 |
| UBE2C | ubiquitin conjugating enzyme E2 C | 28 |
| HNRNPC | heterogeneous nuclear ribonucleoprotein C | 26 |
| LSM2 | LSM2 homolog, U6 small nuclear RNA and mRNA degradation associated | 25 |
| EFTUD2 | elongation factor Tu GTP binding domain containing 2 | 25 |
| RPP38 | ribonuclease P/MRP subunit p38 | 24 |
| CPSF3 | cleavage and polyadenylation specific factor 3 | 24 |

Supplementary Table 6. DEGs associated with both specific miRNA and DNA methylation CpG sites between GDM and healthy samples.

| **Gene** | **DNA Methylation** | | | | **miRNA** | |
| --- | --- | --- | --- | --- | --- | --- |
|  | **cg ID** | **Chr** | **Site** | **Relation to CpG Island** | **miR** | **Binding site** |
| ***Up-regulated genes affected by both low miRNA and hypomethylation*** | | | | | | |
| ABR | cg04478251 | 17 | Body |  | hsa-miR-145-5p | CDS |
|  | cg04724319 | 17 | Body | N_Shelf |  |  |
|  | cg14236443 | 17 | Body |  |  |  |
|  | cg17208740 | 17 | Body | N_Shore |  |  |
|  | cg23283335 | 17 | Body | S_Shore |  |  |
| ACAP3 | cg18565510 | 1 | Body | N_Shelf | hsa-miR-31-5p | CDS |
|  | cg19414302 | 1 | Body |  |  |  |
|  | cg27185793 | 1 | Body | N_Shelf |  |  |
| ADCY7 | cg03485674 | 16 | Body |  | hsa-miR-24-3p | CDS |
|  | cg06897661 | 16 | 5'UTR |  |  |  |
|  | cg10341242 | 16 | Body |  |  |  |
| ADD1 | cg12655250 | 4 | 5'UTR |  | hsa-miR-654-5p | 5UTR |
| AFAP1 | cg00135399 | 4 | Body |  | hsa-miR-654-5p | 3UTR |
|  | cg06934132 | 4 | Body | Island |  |  |
|  | cg07109358 | 4 | Body | Island |  |  |
|  | cg10250107 | 4 | 5'UTR | Island |  |  |
|  | cg22430985 | 4 | Body |  |  |  |
|  | cg25003275 | 4 | 5'UTR | N_Shore |  |  |
| ALS2 | cg01813165 | 2 | TSS1500 | S_Shore | hsa-miR-145-5p | CDS |
| ANKMY1 | cg07592361 | 2 | 5'UTR | Island | hsa-miR-24-3p | CDS |
|  | cg20647656 | 2 | Body |  |  |  |
|  | cg27504117 | 2 | TSS1500 | N_Shore |  |  |
| APBB2 | cg11382761 | 4 | Body |  | hsa-miR-31-5p, hsa-miR-134-5p, hsa-miR-24-3p | CDS, 3UTR, 3UTR |
| B3GNT9 | cg06889547 | 16 | 3'UTR | N_Shore | hsa-miR-134-5p | 3UTR |
| BNC2 | cg23617327 | 9 | TSS1500 | Island | hsa-miR-134-5p | CDS |
| CLTCL1 | cg03339016 | 22 | Body | Island | hsa-miR-24-3p | CDS |
| CYTH2 | cg00718142 | 19 | Body | N_Shelf | hsa-miR-24-3p | CDS |
| DCHS1 | cg05550420 | 11 | 5'UTR |  | hsa-miR-24-3p | CDS |
|  | cg26241462 | 11 | TSS1500 | S_Shore |  |  |
| DDX6 | cg03426840 | 11 | 5'UTR | Island | hsa-miR-152-3p | 3UTR |
| DICER1 | cg01049870 | 14 | 5'UTR |  | hsa-miR-196b-5p | CDS |
| DKK3 | cg13024368 | 11 | 1stExon | Island | hsa-miR-151a-5p | CDS |
|  | cg22082397 | 11 | TSS1500 | S_Shore |  |  |
|  | cg26446832 | 11 | Body |  |  |  |
| DNMBP | cg18805518 | 10 | 5'UTR |  | hsa-miR-151a-5p | CDS |
|  | cg19212550 | 10 | 5'UTR |  |  |  |
| EPHB2 | cg01970575 | 1 | TSS1500 | N_Shore | hsa-miR-24-3p | CDS |
|  | cg12046053 | 1 | Body |  |  |  |
| FBN1 | cg17835606 | 15 | Body |  | hsa-miR-152-3p | 3UTR |
| FEM1B | cg02389877 | 15 | 3'UTR |  | hsa-miR-31-5p | 3UTR |
| FER | cg16055106 | 5 | Body |  | hsa-miR-31-5p | CDS |
| FNDC3B | cg12408494 | 3 | TSS1500 | N_Shore | hsa-miR-134-5p | 3UTR |
|  | cg17558268 | 3 | Body |  |  |  |
|  | cg25668416 | 3 | 5'UTR | Island |  |  |
| GLG1 | cg02899960 | 16 | TSS1500 | S_Shore | hsa-miR-152-3p | 3UTR |
| IGF1R | cg09824900 | 15 | Body |  | hsa-miR-145-5p | 3UTR |
|  | cg20479870 | 15 | TSS200 | Island |  |  |
|  | cg11544420 | 15 | Body |  |  |  |
|  | cg12402183 | 15 | Body |  |  |  |
|  | cg15849465 | 15 | Body | N_Shelf |  |  |
|  | cg20239485 | 15 | Body |  |  |  |
|  | cg20388729 | 15 | Body |  |  |  |
| IGFBP5 | cg03222971 | 2 | Body | N_Shore | hsa-miR-24-3p | CDS |
| KANK2 | cg13516362 | 19 | TSS1500 | S_Shore | hsa-miR-145-5p | 3UTR |
|  | cg26878688 | 19 | TSS1500 | S_Shore |  |  |
| KCNJ6 | cg05434312 | 21 | 5'UTR | N_Shore | hsa-miR-654-5p | 5UTR |
|  | cg07827943 | 21 | TSS1500 | Island |  |  |
| KLF6 | cg24287110 | 10 | Body | S_Shore | hsa-miR-152-3p, hsa-miR-24-3p, hsa-miR-656-3p | 3UTR, 3UTR, 3UTR |
| LASP1 | cg03322670 | 17 | TSS200 | Island | hsa-miR-145-5p, hsa-miR-31-5p | 3UTR, 3UTR |
| LIMD1 | cg04037228 | 3 | 1stExon | S_Shore | hsa-miR-24-3p | 3UTR |
| LMBR1L | cg21747160 | 12 | TSS1500 | S_Shore | hsa-miR-24-3p | 3UTR |
| MACF1 | cg00426822 | 1 | TSS1500 | N_Shore | hsa-miR-369-5p | 3UTR |
| MAP1B | cg05528102 | 5 | Body | Island | hsa-miR-31-5p | CDS |
|  | cg21912162 | 5 | Body |  |  |  |
| MAST3 | cg18335931 | 19 | Body | Island | hsa-miR-24-3p | CDS |
| MEF2D | cg26885488 | 1 | 5'UTR |  | hsa-miR-654-5p | 3UTR |
| NAV2 | cg04916810 | 11 | Body | N_Shore | hsa-miR-196b-5p | CDS |
|  | cg05491166 | 11 | Body |  | hsa-miR-24-3p | CDS |
|  | cg10281394 | 11 | 3'UTR |  |  |  |
|  | cg12859429 | 11 | Body |  |  |  |
|  | cg23944586 | 11 | Body |  |  |  |
|  | cg25607321 | 11 | 5'UTR | Island |  |  |
| NEDD9 | cg27512974 | 6 | 3'UTR |  | hsa-miR-145-5p, hsa-miR-24-3p | 3UTR, 3UTR |
| NEK6 | cg14385362 | 9 | TSS1500 |  | hsa-miR-24-3p | 3UTR |
| NIN | cg08572565 | 14 | TSS1500 | S_Shore | hsa-miR-145-5p | CDS |
|  | cg10169539 | 14 | 5'UTR | N_Shore |  |  |
| NTN4 | cg03100449 | 12 | TSS1500 | S_Shore | hsa-miR-145-5p | CDS |
|  | cg24790471 | 12 | TSS1500 | S_Shore |  |  |
|  | cg26584545 | 12 | Body | N_Shore |  |  |
| NUFIP2 | cg21583412 | 17 | TSS1500 | Island | hsa-miR-145-5p | 3UTR |
| PDE5A | cg19191984 | 4 | TSS1500 | Island | hsa-miR-152-3p | CDS |
| PLK3 | cg04008821 | 1 | TSS1500 | N_Shore | hsa-miR-24-3p | CDS |
| PODXL | cg00186141 | 7 | TSS1500 | Island | hsa-miR-145-5p | 3UTR |
|  | cg02051077 | 7 | 5'UTR | Island |  |  |
|  | cg09859034 | 7 | Body |  |  |  |
|  | cg16657340 | 7 | TSS1500 | Island |  |  |
| POU2F1 | cg05790989 | 1 | 5'UTR |  | hsa-miR-24-3p, hsa-miR-31-5p, hsa-miR-654-5p | CDS, CDS, 3UTR |
|  | cg21182322 | 1 | TSS1500 | Island |  |  |
| PPP3CA | cg09444818 | 4 | Body |  | hsa-miR-145-5p | CDS |
|  | cg12345953 | 4 | Body |  |  |  |
| PTBP2 | cg08462292 | 1 | Body | S_Shore | hsa-miR-196b-5p | CDS |
|  | cg17985656 | 1 | TSS1500 | N_Shore |  |  |
| RANBP2 | cg08028004 | 2 | 1stExon | Island | hsa-miR-145-5p | CDS |
| RNF213 | cg02726136 | 17 | Body |  | hsa-miR-151a-5p | CDS |
|  | cg06188160 | 17 | Body |  |  |  |
| RREB1 | cg04208750 | 6 | 5'UTR | N_Shelf | hsa-miR-145-5p | 3UTR |
|  | cg08651674 | 6 | 5'UTR | Island |  |  |
|  | cg11433624 | 6 | 5'UTR |  |  |  |
|  | cg19113030 | 6 | TSS1500 | Island |  |  |
| RUFY2 | cg02249930 | 10 | Body | Island | hsa-miR-152-3p | CDS |
| SERPINE1 | cg12584355 | 7 | Body |  | hsa-miR-654-5p | CDS |
|  | cg25826546 | 7 | TSS1500 |  |  |  |
| SH3BP4 | cg06638913 | 2 | 5'UTR |  | hsa-miR-654-5p | CDS |
|  | cg12214003 | 2 | TSS1500 | N_Shore |  |  |
|  | cg20553016 | 2 | Body | N_Shore |  |  |
| SPATA13 | cg00967013 | 13 | 5'UTR | S_Shore | hsa-miR-24-3p | CDS |
|  | cg07160044 | 13 | 5'UTR | Island |  |  |
| SPATS2 | cg25274881 | 12 | 5'UTR | S_Shore | hsa-miR-145-5p | 5UTR |
| TACC1 | cg04049530 | 8 | Body |  | hsa-miR-134-5p | 3UTR |
|  | cg11241541 | 8 | 1stExon |  |  |  |
| TET2 | cg09381178 | 4 | TSS1500 | N_Shore | hsa-miR-145-5p, hsa-miR-152-3p | 3UTR, 3UTR |
| TNS1 | cg01138706 | 2 | 5'UTR |  | hsa-miR-24-3p | 3UTR |
|  | cg03323067 | 2 | 5'UTR |  |  |  |
|  | cg12568756 | 2 | Body |  |  |  |
|  | cg18525582 | 2 | TSS1500 |  |  |  |
|  | cg20697464 | 2 | 5'UTR |  |  |  |
|  | cg26638069 | 2 | 5'UTR |  |  |  |
| TPM3 | cg14835484 | 1 | Body | S_Shelf | hsa-miR-145-5p | 3UTR |
|  | cg24546083 | 1 | TSS1500 | Island |  |  |
| TRAK2 | cg24749970 | 2 | 5'UTR | Island | hsa-miR-152-3p | CDS |
| VCPIP1 | cg14249120 | 8 | TSS200 | Island | hsa-miR-24-3p | 3UTR |
| ZBTB20 | cg06225294 | 3 | 5'UTR |  | hsa-miR-31-5p, hsa-miR-145-5p, hsa-miR-376c-3p | CDS, 3UTR, 3UTR |
|  | cg22953510 | 3 | 5'UTR |  |  |  |
| ZMAT3 | cg14983148 | 3 | 5'UTR | Island | hsa-miR-24-3p | CDS |
|  | cg18380974 | 3 | TSS1500 | S_Shore |  |  |
|  | cg26085197 | 3 | TSS200 | Island |  |  |
| ZNF248 | cg04308323 | 10 | TSS1500 | Island | hsa-miR-24-3p | CDS |
|  | cg12309550 | 10 | Body | N_Shelf | hsa-miR-24-3p | 5UTR |
|  | cg15143799 | 10 | TSS200 | Island |  |  |
|  | cg15531788 | 10 | TSS200 | Island |  |  |
| ZNF264 | cg02779761 | 19 | TSS1500 | N_Shore | hsa-miR-24-3p | CDS |
|  | cg08047939 | 19 | TSS1500 | N_Shore | hsa-miR-376c-3p | 3UTR |
|  | cg17670646 | 19 | TSS1500 | N_Shore |  |  |
|  | cg20715764 | 19 | TSS200 | Island |  |  |
|  | cg24716275 | 19 | TSS1500 | N_Shore |  |  |
| ZNF418 | cg26671652 | 19 | 5'UTR | N_Shore | hsa-miR-654-5p | 3UTR |
| ZXDC | cg13264183 | 3 | TSS1500 | Island | hsa-miR-134-5p | CDS |
|  | cg19442647 | 3 | TSS200 | Island | hsa-miR-24-3p | CDS |
| ***Down-regulated genes affected by both high miRNA and hypermethylation*** | | | | | | |
| ABCE1 | cg01713095 | 4 | 3'UTR |  | hsa-miR-30b-3p | 5UTR |
| ANKRD46 | cg03686887 | 8 | TSS1500 | S_Shore | hsa-miR-378a-5p | CDS |
| ANP32E | cg02340851 | 1 | Body | N_Shore | hsa-miR-378c | 3UTR |
|  | cg18878095 | 1 | Body | Island |  |  |
|  | cg21285133 | 1 | TSS1500 | S_Shore |  |  |
| ATG7 | cg01796438 | 3 | TSS1500 | N_Shore | hsa-miR-301a-5p | 3UTR |
|  | cg05643613 | 3 | Body |  |  |  |
|  | cg05672923 | 3 | Body | Island |  |  |
| CDC14A | cg00934037 | 1 | Body |  | hsa-miR-30b-3p | CDS |
|  | cg19083659 | 1 | Body |  |  |  |
| CDCA7L | cg17958283 | 7 | Body |  | hsa-miR-378c | 3UTR |
| CHID1 | cg07066326 | 11 | 5'UTR | N_Shore | hsa-miR-30b-3p | CDS |
|  | cg15082648 | 11 | Body | N_Shelf |  |  |
|  | cg23202388 | 11 | 1stExon | S_Shelf |  |  |
| EFCAB2 | cg03889876 | 1 | Body | Island | hsa-miR-30b-3p | 3UTR |
|  | cg16678437 | 1 | TSS1500 | Island |  |  |
| EPS15L1 | cg21067775 | 19 | Body | Island | hsa-miR-30b-3p | 3UTR |
| FAM110B | cg01602153 | 8 | 5'UTR | Island | hsa-miR-15b-3p | 3UTR |
|  | cg14012365 | 8 | Body | Island |  |  |
|  | cg19502867 | 8 | TSS200 | Island |  |  |
| GART | cg13617782 | 21 | TSS1500 | S_Shore | hsa-miR-30b-3p | CDS |
|  | cg21135948 | 21 | TSS1500 | Island |  |  |
|  | cg22542663 | 21 | TSS1500 | Island |  |  |
| GTPBP10 | cg11909310 | 7 | TSS1500 | Island | hsa-miR-30b-3p | 3UTR |
| HAUS2 | cg05110071 | 15 | Body | S_Shore | hsa-miR-30b-3p | 3UTR |
|  | cg10327976 | 15 | Body | S_Shelf |  |  |
| LYRM4 | cg11983857 | 6 | Body |  | hsa-miR-30b-3p | 3UTR |
| METTL1 | cg25452172 | 12 | 3'UTR | N_Shelf | hsa-miR-30b-3p | CDS |
| MTDH | cg00265490 | 8 | TSS1500 | N_Shore | hsa-miR-378a-5p | CDS |
|  | cg08874788 | 8 | TSS200 | Island |  |  |
|  | cg25544073 | 8 | TSS200 | Island |  |  |
| NFIC | cg07818063 | 19 | Body | N_Shelf | hsa-miR-30b-3p | CDS |
|  | cg08380311 | 19 | Body | Island |  |  |
|  | cg09422806 | 19 | Body | N_Shelf |  |  |
|  | cg17887593 | 19 | TSS1500 | Island |  |  |
| NOSTRIN | cg24861006 | 2 | 5'UTR |  | hsa-miR-301a-5p | 3UTR |
| NUP210 | cg03931678 | 3 | TSS1500 | Island | hsa-miR-99a-3p | CDS |
| PANK4 | cg23665250 | 1 | Body | Island | hsa-miR-378a-5p | CDS |
| PLA2G4A | cg09320113 | 1 | 5'UTR |  | hsa-miR-33a-3p | CDS |
|  | cg24863175 | 1 | Body |  |  |  |
| PLAGL2 | cg18463417 | 20 | TSS1500 | S_Shore | hsa-miR-30b-3p | 3UTR |
| POLR2D | cg00606472 | 2 | TSS200 | Island | hsa-miR-30b-3p | 3UTR |
|  | cg01200177 | 2 | TSS1500 | S_Shore |  |  |
|  | cg19566038 | 2 | TSS1500 | Island |  |  |
| PRKAA2 | cg09313917 | 1 | Body | S_Shore | hsa-miR-301b-3p | 3UTR |
|  | cg09935045 | 1 | Body | Island |  |  |
| PRPF18 | cg05686950 | 10 | Body |  | hsa-miR-33a-3p | CDS |
|  | cg17601595 | 10 | TSS200 | N_Shore |  |  |
| PSMA5 | cg04240373 | 1 | TSS1500 | S_Shore | hsa-miR-33a-3p | 5UTR |
| RDH10 | cg27596828 | 8 | 3'UTR |  | hsa-miR-30b-3p | 3UTR |
| RFC5 | cg14967768 | 12 | 5'UTR | S_Shore | hsa-miR-4521 | CDS |
|  | cg23343264 | 12 | TSS1500 | N_Shore |  |  |
| RNF114 | cg08659621 | 20 | TSS200 | Island | hsa-miR-30b-3p | 3UTR |
|  | cg26748394 | 20 | Body | S_Shelf |  |  |
| SKA1 | cg20857301 | 18 | TSS1500 | N_Shore | hsa-miR-30b-3p | CDS |
| SNAP29 | cg06723301 | 22 | 1stExon | Island | hsa-miR-345-5p | 5UTR |
|  | cg08524698 | 22 | TSS200 | Island |  |  |
| SRP19 | cg01255486 | 5 | Body | S_Shelf | hsa-miR-378c | CDS |
|  | cg15551292 | 5 | TSS200 | Island |  |  |
| SRP68 | cg08510930 | 17 | 3'UTR |  | hsa-miR-4521 | CDS |
| STAMBP | cg02352181 | 2 | Body | S_Shore | hsa-miR-378a-5p | 3UTR |
| STIM2 | cg13621396 | 4 | TSS1500 | N_Shore | hsa-miR-188-5p | CDS |
| STT3A | cg02921249 | 11 | Body |  | hsa-miR-188-5p | CDS |
|  |  |  |  |  | hsa-miR-4521 | CDS |
| TIPIN | cg08246659 | 15 | Body |  | hsa-miR-4521 | CDS |
| TNFRSF9 | cg23959705 | 1 | TSS1500 | N_Shore | hsa-miR-30b-3p | 3UTR |
|  |  |  |  |  | hsa-miR-378c | 3UTR |
|  |  |  |  |  | hsa-miR-378a-5p | CDS |
| TTLL4 | cg11123644 | 2 | 5'UTR |  | hsa-miR-4521 | CDS |
|  | cg27629986 | 2 | 5'UTR |  |  |  |
| UBE2I | cg03887008 | 16 | 5'UTR | S_Shore | hsa-miR-188-5p | 3UTR |
|  | cg04688590 | 16 | Body | S_Shore |  |  |
|  | cg06599546 | 16 | 5'UTR | S_Shore |  |  |
|  | cg16569063 | 16 | 3'UTR |  |  |  |
| XPO4 | cg07024905 | 13 | Body |  | hsa-miR-345-5p | 3UTR |
|  |  |  |  |  | hsa-miR-30b-3p | CDS |
| ZDHHC3 | cg01367339 | 3 | Body |  | hsa-miR-30b-3p | 3UTR |
|  |  |  |  |  | hsa-miR-378a-5p | 3UTR |
| ZFP64 | cg14588259 | 20 | Body | S_Shore | hsa-miR-30b-3p | 3UTR |
|  | cg23604151 | 20 | Body | N_Shelf |  |  |
| ZNF619 | cg03053581 | 3 | Body | S_Shelf | hsa-miR-30b-3p | CDS |
| **C11orf49#** | cg03339077 | 11 | Body |  | hsa-miR-378c | 3UTR |
|  | cg03522150 | 11 | Body |  |  |  |
|  | cg23072973 | 11 | 3'UTR |  |  |  |
| **C15orf39#** | cg03980226 | 15 | 3'UTR | S_Shelf | hsa-miR-188-5p | CDS |
| **C19orf47#** | cg11432751 | 19 | TSS200 | Island | hsa-miR-30b-3p | 3UTR |
|  | cg17508018 | 19 | TSS200 | Island |  |  |
| **RBBP4#** | cg13641837 | 1 | TSS200 | Island | hsa-miR-301a-5p | 3UTR |
|  | cg24463320 | 1 | Body | S_Shelf | hsa-miR-378a-5p | 3UTR |

#The four gene were not identified in MERAV online database.

Supplementary Figure. Binding mode of representative trichostatin A and proteins. (A). ATG7. (B). DICER1. (C). IGF1R. (D). RANBP2. The red amino acids indicate that they bind to the drug and the yellow dotted line represents hydrogen bonds.
